# Identification of novel ligands interacting with kappa opioid receptors

**DOI:** 10.1186/1471-2210-11-S2-A15

**Published:** 2011-09-05

**Authors:** Mariana Spetea, Muhammad F Asim, Ilona P Berzetei-Gurske, Gerhard Wolber, Helmut Schmidhammer

**Affiliations:** 1 Department of Pharmaceutical Chemistry, Institute of Pharmacy and Center for Molecular Biosciences, University of Innsbruck, 6020 Innsbruck, Austria; 2 Biosciences Division, SRI International, Menlo Park, CA 94025, USA; 3 Department of Pharmaceutical Chemistry, Institute of Pharmacy, Free University of Berlin, 14195 Berlin, Germany

## Background

The κ opioid (KOP) receptor belongs to the family of seven transmembrane G protein-coupled receptors (GPCRs) and it plays a significant role in a broad range of physiological functions. Stimulation of the KOP receptor results in analgesic actions, and KOP agonists appear to have some advantages over the μ opioid (MOP) receptor agonists. Inhibiting KOP receptors is proposed to be useful for treating addiction and stress-related conditions, such as depression and anxiety. The pharmacology of currently available KOP antagonists shows a delay in onset of action and an extremely long duration of action *in vivo*, which might limit their therapeutic application. The search for new ligands with potent biological activities, particularly as potential novel therapeutic agents, utilizing computational and synthetical approaches, is a key goal of life science research and drug development. Herein, we present the *in silico*, *in vitro* and *in vivo* profiles of new molecular scaffolds as novel KOP receptor ligands.

## Methods

LigandScout and Catalyst softwares were used to generate and validate a merged feature ligand-based 3D pharmacophore model for KOP receptors. Biological activities were evaluated in *in vitro* opioid receptor binding and [^35^S]GTPγS functional assays. Antinociceptive properties were assessed in mice using the writhing test.

## Results

The integrated computational screening strategy has led to the discovery of sewarine as KOP receptor ligand. This phenolic alkaloid from the plant *Rhazya stricta* binds with high selectivity to the KOP receptor and shows antagonist activity. A comprehensive SAR analysis on several analogues was pursued and primary chemical features responsible for KOP activity have been identified. Combining synthetical and pharmacological methodologies, two phenolic molecules were identified as novel highly selective ligands interacting with KOP receptors, and displaying full agonist or partial agonist properties, respectively. Dose-dependent and nor-BNI-sensitive antinociceptive effects were produced by the KOP agonist after subcutaneous administration to mice, exhibiting a potency comparable to that of U-50,488.

## Conclusions

This study uncovers new classes of ligands interacting with KOP receptors and sharpens the understanding of ligand-receptor interactions, thus increasing the chance of developing useful clinical agents among KOP ligands.

